# Mechanisms of Doxorubicin-Induced Cardiac Senescence and Potential Therapeutic Strategies

**DOI:** 10.3390/biom16081180

**Published:** 2026-08-12

**Authors:** Yanli Bai, Wen Yang, Zirong Wang, Qianqian Yang, Zhongping Zhang, Jialong Liu, Yafang Qi, Dongling Liu

**Affiliations:** 1 School of Basic Medical Sciences, Gansu University of Chinese Medicine, Lanzhou 730000, China; baiyl@gszy.edu.cn; 2 School of Pharmacy, Gansu University of Chinese Medicine, Lanzhou 730000, China; 3 School of Bioengineering and Technology, Tianshui Normal University, Tianshui 741000, China; 20250902020@st.tsnu.edu.cn; 4 Gansu Pharmaceutical Industry Innovation Research Institute, Gansu University of Chinese Medicine, Lanzhou 730000, China; 5 Northwest Collaborative Innovation Center for Traditional Chinese Medicine, Lanzhou 730000, China

**Keywords:** cardiac senescence, doxorubicin, cardiotoxicity, senescence-targeted therapy

## Abstract

Doxorubicin (DOX) is a widely used anthracycline chemotherapeutic agent; however, its clinical application is limited by dose-dependent cardiotoxicity, which can result in progressive cardiac dysfunction and heart failure. Increasing evidence indicates that DOX-induced cardiotoxicity is closely associated with premature cardiac senescence, a pathological process distinct from physiological cardiac aging. DOX induces senescence-associated alterations in multiple cardiac cell populations, disrupting cardiac homeostasis and contributing to pathological remodeling. In this review, we summarize current advances in DOX-induced cardiac senescence, focusing on the contributions of different cardiac cell types, the underlying molecular mechanisms, and emerging therapeutic strategies. We further discuss the challenges and future perspectives for developing effective interventions that alleviate cardiac senescence while preserving the anticancer efficacy of DOX. Understanding the mechanisms driving DOX-induced cardiac senescence may provide new opportunities to develop effective cardioprotective strategies and improve long-term cardiac outcomes after chemotherapy.

## 1. Introduction

Oncological diseases are among the most prevalent public health concerns and rank as the second leading cause of death, following cardiovascular diseases. Doxorubicin (DOX), commonly known as Adriamycin, is a widely used anthracycline antibiotic and a chemotherapy agent. It has shown therapeutic efficacy in treating a range of cancers, including breast, lung, leukemia, and colon cancers [1]. However, its clinical application is limited by dose-dependent cardiotoxicity, which may result in progressive cardiac dysfunction and heart failure. Although oxidative stress, mitochondrial dysfunction, DNA damage, calcium dysregulation, and apoptosis are recognized contributors to DOX-induced cardiotoxicity, these mechanisms alone cannot fully explain the persistent and delayed cardiac deterioration following chemotherapy [2].

Increasing evidence indicates that DOX-induced cardiotoxicity is closely associated with premature cardiac senescence, representing an accelerated pathological process distinct from physiological cardiac senescence. Unlike physiological cardiac senescence, which develops gradually through lifelong accumulation of genomic instability, mitochondrial decline, metabolic alterations, and chronic low-grade inflammation, DOX-induced cardiac senescence is initiated by chemotherapy-associated cellular injury, particularly DNA damage and mitochondrial stress (Table 1). Cellular senescence is characterized by stable cell cycle arrest, persistent stress responses, metabolic alterations, and acquisition of the senescence-associated secretory phenotype (SASP) [3]. Following DOX exposure, persistent DNA damage responses, mitochondrial dysfunction, and inflammatory activation promote senescent phenotypes and impair cardiac homeostasis [4,5].

Importantly, DOX-induced cardiac senescence is a multicellular pathological process rather than a cardiomyocyte-specific event. In addition to cardiomyocytes, cardiac fibroblasts, endothelial cells, vascular smooth muscle cells, cardiac progenitor/stem cells, and immune cells contribute to senescence-associated remodeling through altered intercellular communication, inflammatory signaling, extracellular matrix remodeling, and impaired tissue repair [6,7,8]. Thus, understanding cell-type-specific responses and interactions within the cardiac microenvironment is essential for elucidating the complexity of DOX-induced cardiac senescence.

At the molecular level, DOX-induced cardiac senescence is regulated by interconnected pathways, including oxidative stress, telomere dysfunction, mitochondrial impairment, SASP-associated inflammatory signaling, non-coding RNA regulation, autophagy disruption, and apoptosis [9,10,11]. Although previous reviews have extensively summarized conventional mechanisms of DOX cardiotoxicity, an integrated overview focusing specifically on DOX-induced cardiac senescence, including its cellular origins, molecular mechanisms, and therapeutic implications, remains limited.

**Figure 1:**
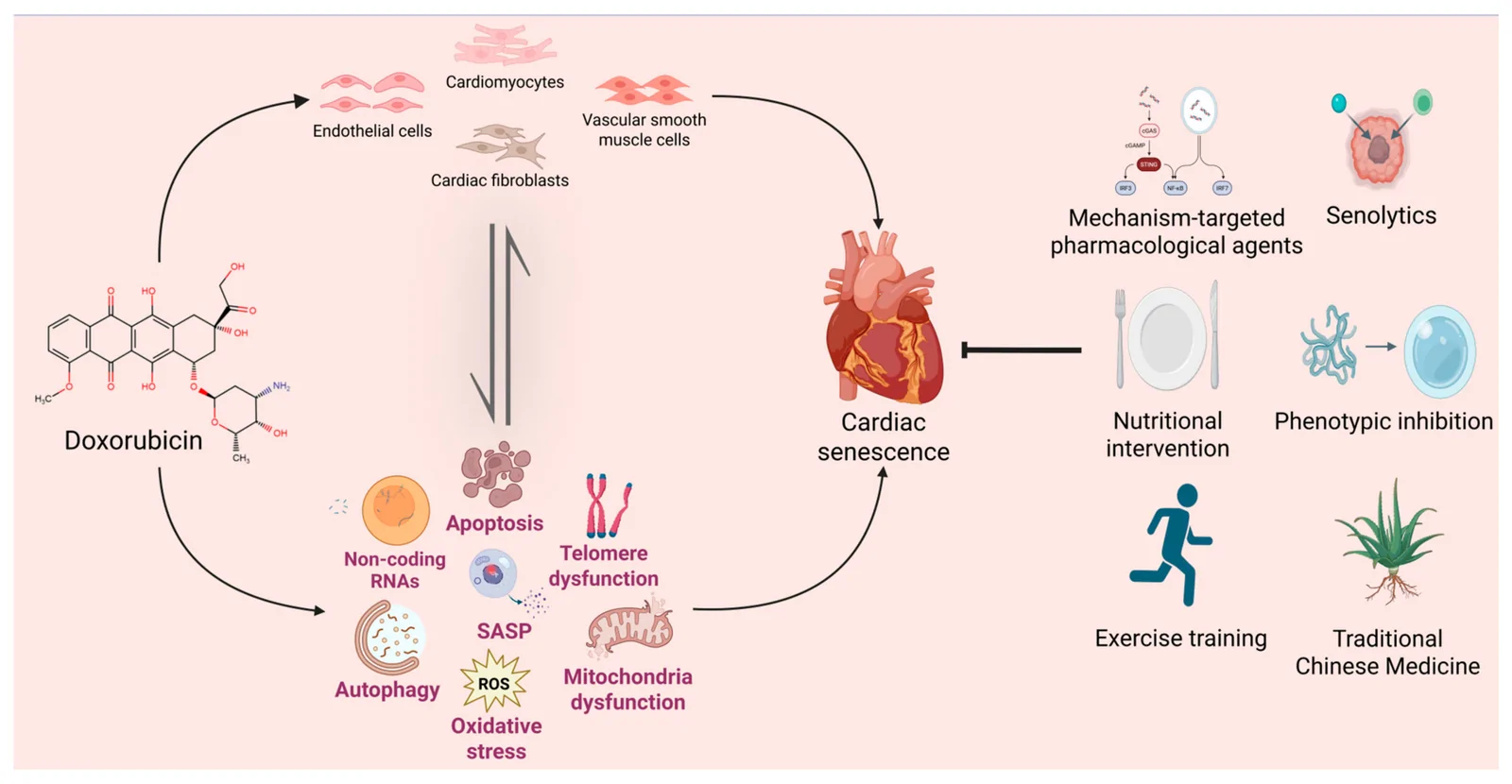
DOX-induced cardiac senescence: target cells, mechanisms, and countermeasures. Doxorubicin targets multiple cardiac cell populations, including cardiomyocytes, cardiac fibroblasts, endothelial cells, and vascular smooth muscle cells, triggering senescence-related mechanisms, including oxidative stress, mitochondrial dysfunction, telomere dysfunction, senescence-associated secretory phenotype (SASP), non-coding RNA regulation, autophagy, and apoptosis, which collectively promote cellular senescence, cardiac dysfunction, and pathological remodeling. Potential countermeasures to mitigate this senescence progression include mechanism-targeted pharmacological agents, nutritional intervention, exercise training, senolytic intervention, senescent phenotypic inhibition, and Traditional Chinese Medicine. Solid arrows indicate DOX-induced pathological progression, whereas the blunt-ended line indicates the inhibitory effects of the intervention strategies on DOX-induced cardiac senescence. Created in BioRender. Zhang, Z. (2026) https://BioRender.com/um2swv0.

In this review, we summarize current advances in DOX-induced cardiac senescence by discussing the contributions of different cardiac cell populations, the underlying molecular mechanisms, and emerging intervention strategies, including nutritional intervention, exercise training, mechanism-targeted pharmacological agents, senescence-targeted therapies, and Traditional Chinese Medicine (Figure 1).

**Table 1:** Mechanistic distinctions between physiological cardiac senescence and doxorubicin-induced premature cardiac senescence.

Feature	Physiological Cardiac Senescence	Doxorubicin-Induced Premature Cardiac Senescence
Initiating stimulus	Chronological aging with cumulative genomic instability, mitochondrial decline, metabolic alterations, and chronic low-grade inflammation	Two major DOX-specific toxic stimuli: TOP2B inhibition triggering massive DNA double-strand breaks [12,13,14]; cationic DOX directly binds mitochondrial cardiolipin to disrupt membrane structure and function [15]
Time course	Progressive process developing over years or decades	Cardiac senescence-associated alterations may emerge within weeks to months after DOX exposure in experimental models [16,17]
DNA damage	Slow lifelong telomere erosion acts as the dominant initiator of the DNA damage response (DDR) [18]	Doxorubicin initiates massive acute DNA double-strand breaks via TOP2B inhibition; telomere attrition is a secondary downstream consequence of DOX-mediated injury [7,19]
Mitochondrial injury	Gradual, mild cristae deterioration and slow reactive oxygen species (ROS) elevation over the lifespan [20,21,22]	Acute DOX–cardiolipin complex formation rapidly destroys mitochondrial cristae architecture, triggering a massive acute ROS burst [23,24]
Immune regulation	Chronic inflammaging is associated with age-related alterations in immune homeostasis that favor the persistence of senescent cells and prolonged inflammatory signaling [25,26,27]	DOX disrupts macrophage recruitment and polarization, impairing inflammatory resolution and myocardial repair; restoration of macrophage function attenuates cardiac injury [28]
Consequences for tissue remodeling	Persistent SASP gradually remodels the myocardial microenvironment, contributing to progressive extracellular matrix remodeling and functional decline during aging [29]	Persistent SASP together with impaired macrophage-mediated repair accelerates maladaptive myocardial remodeling following DOX exposure [28,30]

DOX-induced cardiac senescence shares several hallmark features with physiological cardiac senescence, including oxidative stress, mitochondrial dysfunction, DNA damage responses, and chronic inflammatory signaling. However, these processes arise from fundamentally different initiating events. Physiological cardiac senescence develops gradually through lifelong accumulation of endogenous stress, whereas doxorubicin induces premature senescence following chemotherapy-associated cellular injury. Therefore, this review primarily focuses on mechanistic and therapeutic evidence derived directly from experimental and clinical studies of DOX-induced cardiac senescence. Findings from physiological cardiac senescence are discussed only when they provide mechanistic insight into conserved biological processes or help explain observations that remain incompletely characterized in DOX models.

## 2. Cell Types Involved in DOX-Induced Cardiac Senescence

Cellular senescence is a heterogeneous process characterized by irreversible cell cycle arrest and context-dependent functional decline [31]. According to the initiating stimuli, cellular senescence can be classified into replicative senescence, oncogene-induced senescence, and stress-induced premature senescence [32]. Although physiological and DOX-induced cardiac senescence arise from distinct initiating mechanisms, they share several conserved features and mechanisms. DOX-induced cardiac senescence represents a specific form of stress-induced premature senescence triggered by chemotherapy-associated cellular injury.

**Figure 2:**
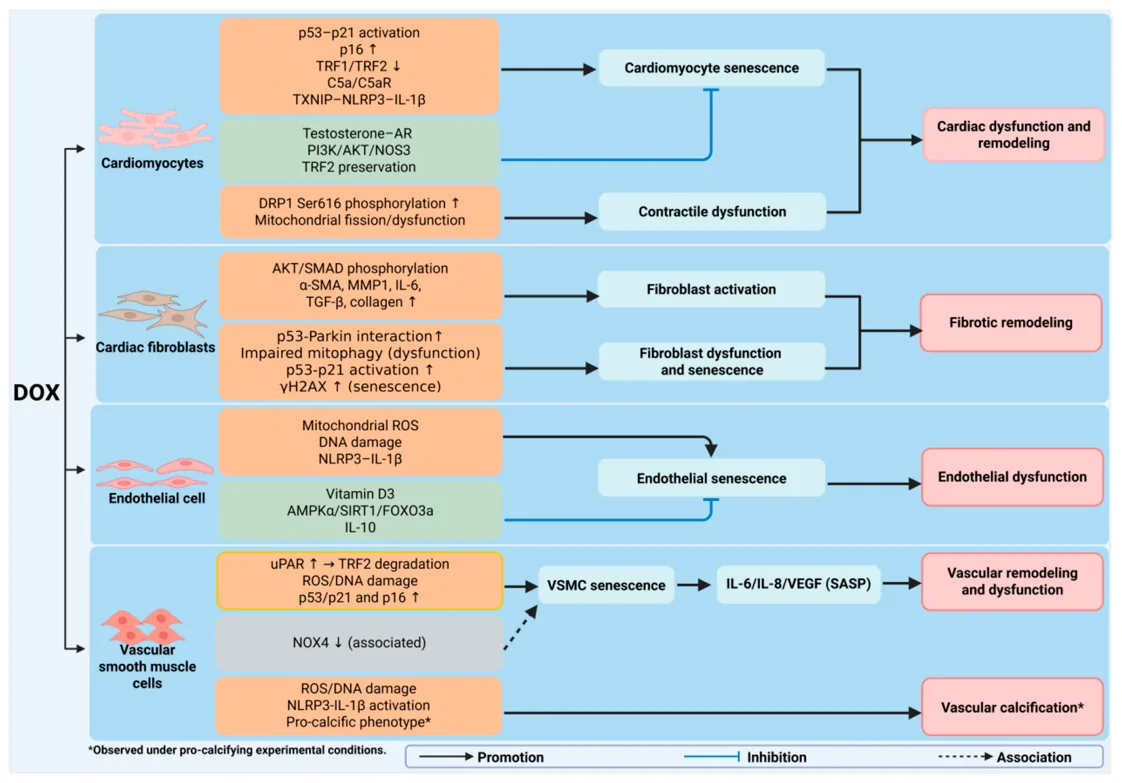
Cell-type-specific mechanisms of DOX-induced cardiac and vascular senescence and dysfunction. In cardiomyocytes, p53-p21 activation, p16 upregulation, reduced TRF1/TRF2 expression, C5a/C5aR signaling, and TXNIP-NLRP3-IL-1β activation promote senescence, whereas DRP1 Ser616 phosphorylation and mitochondrial fission/dysfunction contribute to contractile dysfunction. Testosterone-AR-PI3K/AKT/NOS3 signaling preserves TRF2 and inhibits cardiomyocyte senescence. In cardiac fibroblasts, AKT/SMAD phosphorylation and increased expression of α-SMA, MMP1, IL-6, TGF-β, and collagen promote fibroblast activation, while p53-Parkin interaction, impaired mitophagy, p53-p21 activation, and γH2AX accumulation contribute to fibroblast dysfunction and senescence. In endothelial cells, mitochondrial ROS, DNA damage, and NLRP3-IL-1β signaling promote senescence and endothelial dysfunction, whereas vitamin D3-AMPKα/SIRT1/FOXO3a-IL-10 signaling is protective. In VSMCs, uPAR-dependent TRF2 degradation, ROS/DNA damage, and p53/p21 and p16 activation promote senescence and SASP, whereas NOX4 downregulation is associated with, but has not been established as necessary for, DOX-induced senescence. In a parallel pathway, DOX-induced ROS/DNA damage and NLRP3-IL-1β activation promote a pro-calcific phenotype under pro-calcifying experimental conditions. Created in BioRender. Zhang, Z. (2026) https://BioRender.com/rz970xx.

Doxorubicin induces accelerated cardiac senescence involving multiple cardiac cell types, including cardiomyocytes, fibroblasts, endothelial cells, and other cell populations. The underlying mechanisms vary among different cell types, leading to distinct pathological phenotypes (Figure 2). Here, we summarize the cell types involved in DOX-induced cardiac senescence.

### 2.1. Cardiomyocytes

**Figure 3:**
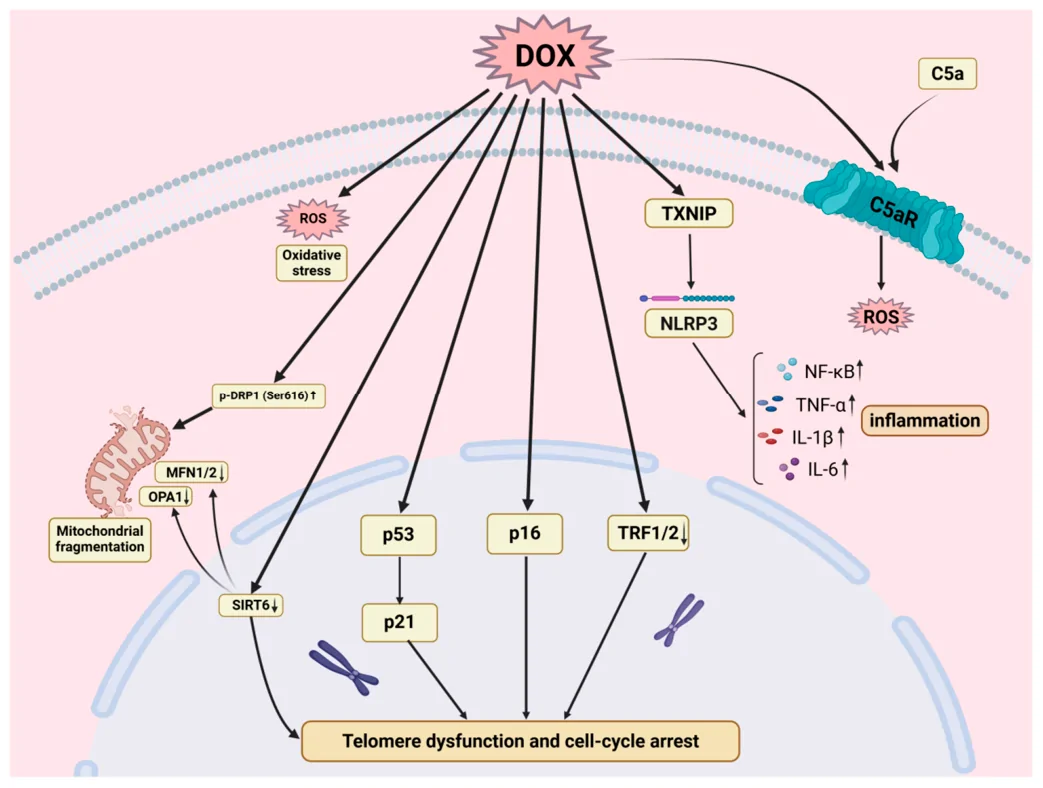
Doxorubicin impairs cardiomyocyte function through three primary pathways: disrupted mitochondrial dynamics, telomere dysfunction and cell-cycle arrest, and oxidative stress and inflammatory signaling. (1) Mitochondrial Dynamics: DOX enhances mitochondrial fission by promoting DRP1 phosphorylation at Ser616. Additionally, DOX-induced downregulation of SIRT6 reduces the expression of the mitochondrial fusion proteins MFN1/2 and OPA1, collectively leading to mitochondrial fragmentation. (2) Telomere Dysfunction and Cell-Cycle Arrest: DOX activates p53–p21 signaling and upregulates p16, leading to cell-cycle arrest. Concurrent downregulation of SIRT6 and the telomere-binding proteins TRF1/2 impairs telomere maintenance and contributes to telomere dysfunction. (3) Oxidative Stress and Inflammatory Signaling: DOX induces oxidative stress by promoting ROS accumulation. It upregulates TXNIP expression and activates the NLRP3 inflammasome. C5a–C5aR signaling further enhances ROS production, accompanied by NF-κB activation and increased TNF-α, IL-1β, and IL-6. Solid black arrows indicate promotion or activation, and “p-” denotes phosphorylation. Created in BioRender. Zhang, Z. (2026) https://BioRender.com/4h8l0rh.

Cardiomyocytes are terminally differentiated cells with limited regenerative capacity and are highly vulnerable to stress-induced senescence (Figure 3). DOX exposure induces a senescence-like phenotype in cardiomyocytes, characterized by increased senescence-associated β-galactosidase activity, elevated cyclin-dependent kinase inhibitors, impaired contractile function, and reduced telomerase activity.

Oxidative stress and DNA damage responses are central events in DOX-induced cardiomyocyte senescence. DOX generates excessive reactive oxygen species (ROS) through redox cycling, leading to activation of stress-responsive pathways [19]. Low-dose DOX promotes premature senescence rather than apoptosis through PML-associated p53 activation and senescence-related cell cycle regulation [19]. In addition, DOX-induced alterations in telomere-binding proteins determine cardiomyocyte fate: reduced TRF1 and TRF2 expression after low-dose DOX exposure causes telomere dysfunction and persistent senescence, whereas higher doses preferentially trigger apoptosis [5]. Testosterone-mediated activation of androgen receptor (AR)–PI3K/AKT/NOS3 signaling was shown to preserve TRF2 expression and attenuate DOX-induced cardiomyocyte senescence, highlighting the importance of telomere maintenance in this process [33].

Mitochondrial dysfunction and stress-responsive signaling contribute to the progression of DOX-induced cardiomyocyte senescence. DOX promotes Drp1-mediated mitochondrial fragmentation, resulting in impaired mitochondrial dynamics and metabolic dysfunction, which may sustain oxidative stress and enhance senescence-associated responses [34]. In addition, DOX activates specific senescence-regulatory pathways, such as lincRNA-p21-mediated Wnt/β-catenin signaling, thereby promoting the establishment of cardiomyocyte senescence and associated molecular changes [35].

Once established, the senescent phenotype can be further amplified and maintained by inflammatory signaling pathways. Activation of the complement C5a/C5aR axis contributes to cardiomyocyte senescence after DOX exposure, whereas C5a receptor inhibition alleviates senescence-associated changes and improves telomere-associated dysfunction [36]. Furthermore, TXNIP-mediated activation of the NLRP3 inflammasome promotes inflammatory responses and senescence phenotypes in DOX-treated cardiomyocytes, while inhibition of TXNIP/NLRP3 signaling attenuates these effects [37].

Persistent accumulation of senescent cardiomyocytes contributes to progressive cardiac dysfunction and late-onset cardiotoxicity after DOX exposure. Long-term DOX treatment accelerates cardiomyocyte senescence, accompanied by increased cardiac mitochondrial DNA damage and senescence-associated markers, indicating that sustained senescence is an important mechanism underlying delayed cardiac dysfunction after chemotherapy [9].

### 2.2. Cardiac Fibroblasts

Cardiac fibroblasts (CFs) are central regulators of extracellular matrix (ECM) homeostasis and cardiac remodeling and actively communicate with cardiomyocytes to coordinate myocardial responses to injury [38,39]. Increasing evidence suggests that CF dysfunction and senescence are important contributors to DOX-induced cardiac remodeling and aging.

Following DOX exposure, cardiac fibroblasts undergo functional activation characterized by fibroblast-to-myofibroblast transition (FMT), accompanied by increased expression of fibrosis-associated genes and inflammatory mediators, thereby promoting extracellular matrix remodeling and myocardial fibrosis [40]. In parallel, DOX-induced DNA damage activates p53 signaling and impairs mitochondrial quality control by preventing Parkin-mediated mitophagy, resulting in mitochondrial dysfunction, reduced proliferative and migratory capacity, and altered expression of remodeling-associated genes in cardiac fibroblasts [41]. These findings indicate that mitochondrial stress and impaired cellular homeostasis are early events underlying cardiac fibroblast dysfunction during DOX-induced cardiotoxicity.

Persistent cellular stress further promotes cardiac fibroblast senescence. DOX-treated cardiac fibroblasts exhibit senescence-associated phenotypes and release extracellular vesicles (EVs), which regulate fibroblast autocrine signaling through AMPKα-dependent modulation of autophagy, highlighting a role for senescent CFs in remodeling the cardiac microenvironment [42]. Moreover, the matricellular protein CCN5 (WISP2) suppresses DOX-induced senescence in cardiac fibroblasts by reducing p53/p21 activation, SA-β-gal activity, and γH2AX accumulation, providing direct evidence that fibroblast senescence contributes to chemotherapy-associated cardiac senescence and represents a potential therapeutic target [43].

### 2.3. Endothelial Cells

Endothelial cells (ECs) play essential roles in maintaining cardiac vascular homeostasis by regulating vascular integrity and endothelial function. Increasing evidence indicates that endothelial senescence is an important mechanism contributing to doxorubicin (DOX)-induced vascular injury and cardiovascular toxicity.

DOX induces endothelial senescence through multiple stress responses, including mitochondrial dysfunction, oxidative stress, DNA damage, and inflammatory activation. In human endothelial cells, DOX exposure increases mitochondrial ROS production and promotes DNA damage and senescence-associated alterations, whereas mitochondrial-targeted antioxidant treatment alleviates these effects, highlighting the contribution of mitochondrial oxidative stress to endothelial senescence [44]. Consistently, EA.hy926 cells and primary human umbilical vein endothelial cells (HUVECs) exhibited similar senescence phenotypes following doxorubicin exposure [45]. Furthermore, DOX activates the NLRP3 inflammasome/IL-1β signaling axis in endothelial cells, promoting inflammatory responses and reinforcing senescence progression, while metformin suppresses SASP secretion and inflammatory activation in DOX-induced senescent endothelial cells [46,47]. Moreover, Vitamin D3 attenuates DOX-induced endothelial senescence through activation of the AMPKα/SIRT1/FoxO3a pathway and upregulation of IL-10, highlighting the involvement of metabolic and anti-inflammatory signaling in endothelial senescence regulation [6].

Importantly, endothelial senescence contributes directly to DOX-induced vascular dysfunction rather than representing merely a molecular marker. In mouse models and human vascular tissues, DOX-induced accumulation of senescent cells is associated with impaired endothelial-dependent vasodilation, reduced nitric oxide bioavailability, and increased mitochondrial oxidative stress, whereas senolytic treatment improves vascular function [48]. These findings establish endothelial senescence as a critical mediator of DOX-associated cardiovascular toxicity.

### 2.4. Vascular Smooth Muscle Cells (VSMCs)

Vascular smooth muscle cells (VSMCs) are essential components of the vascular wall and regulate vascular tone, extracellular matrix homeostasis, and vascular remodeling. Under aging or pathological stress, VSMCs may acquire cellular senescence characterized by irreversible growth arrest, increased senescence-associated markers, and acquisition of a pro-inflammatory phenotype, thereby contributing to vascular dysfunction.

Direct evidence demonstrates that doxorubicin (DOX) induces premature senescence in human VSMCs. DOX-treated VSMCs exhibit increased SA-β-gal activity, reduced proliferative capacity, and cell cycle arrest, with distinct features compared with replicative senescence [49]. Mechanistically, DOX promotes VSMC senescence through telomere dysfunction by inducing uPAR-dependent degradation of the telomere-binding protein TRF2, resulting in impaired telomere maintenance and activation of DNA damage responses [50]. DOX-induced and replicative senescence in VSMCs were accompanied by reduced NOX4 expression. Independent inhibition or silencing of NOX4 was sufficient to induce permanent growth arrest and pro-inflammatory cytokine secretion through a DNA damage response-independent mechanism, indicating a senescent phenotype distinct from that directly induced by DOX. However, whether NOX4 downregulation is causally required for DOX-induced VSMC senescence remains to be established [51].

Senescent VSMCs can further amplify vascular inflammation through SASP secretion and inflammasome activation. Stress-induced NLRP3 inflammasome activation establishes a pro-inflammatory feedback loop in VSMCs and, under pro-calcifying experimental conditions, promotes an IL-1β-dependent pro-calcific phenotype [8]. Collectively, these findings suggest that DOX-induced VSMC senescence, mediated by telomere dysfunction, oxidative stress, and inflammatory activation, may contribute to vascular remodeling and cardiovascular complications following chemotherapy exposure.

### 2.5. Mesenchymal Stem Cells

Resident cardiac mesenchymal stromal/stem cells contribute to cardiac homeostasis and tissue repair through interactions with cardiomyocytes and other cardiac cell populations. However, mesenchymal stromal/stem cells are not generally considered to contribute substantially to functional cardiomyocyte replacement in the adult heart; instead, their potential reparative effects are thought to be mediated predominantly through paracrine signaling [52]. Therefore, senescence of these cells may impair cardiac repair primarily by altering their secretory activity and interactions with surrounding cardiac cells. Although direct evidence concerning DOX-induced senescence in resident cardiac mesenchymal stromal/stem cells remains limited, studies using MSCs derived from other tissues provide relevant mechanistic evidence. Exposure to clinically relevant concentrations of DOX induces senescence-like alterations in human MSCs derived from bone marrow, adipose tissue, and menstrual blood, characterized by increased senescence-associated β-galactosidase activity, reduced proliferation, cell cycle arrest, and accumulation of DNA damage [53]. In addition, DOX-induced MSC senescence is associated with inflammatory activation and enhanced NF-κB signaling, suggesting that chemotherapy exposure may impair MSC-mediated tissue repair through senescence-associated changes in their secretory and inflammatory profiles [54].

Beyond inducing senescence phenotypes, DOX also compromises the functional properties of MSCs. DOX exposure induces DNA damage responses in human MSCs, highlighting the vulnerability of stem/progenitor cell populations to anthracycline-induced genotoxic stress [55]. Although direct evidence linking MSC senescence to DOX-induced cardiac senescence remains limited, impaired MSC viability, proliferation, and paracrine activity may potentially contribute to reduced regenerative capacity and an unfavorable tissue repair environment following chemotherapy-induced cardiac injury. Therefore, MSCs represent not only potential therapeutic targets but also important regulators of cardiac resilience during DOX exposure.

### 2.6. Other Cell Types and Intercellular Regulation of Cardiac Senescence

Beyond cardiomyocytes, fibroblasts, endothelial cells, vascular smooth muscle cells, and mesenchymal stem cells, other cellular populations also contribute to DOX-induced cardiac senescence. Cardiac progenitor cells (CPCs), as endogenous regenerative populations in the heart, are particularly vulnerable to aging and chemotherapy-induced stress. In human hearts from individuals older than 70 years, a substantial proportion of isolated CPCs exhibit senescence-associated features, including DNA damage, altered telomere maintenance, and SASP activation [56]. DOX exposure further reduces CPC survival and proliferative capacity while impairing their regenerative and paracrine functions, potentially limiting their ability to support cardiomyocyte maintenance, angiogenesis, and myocardial repair [57].

In addition to cell-autonomous senescence, accumulating evidence indicates that senescent cardiac cells establish a complex intercellular communication network through the senescence-associated secretory phenotype (SASP). Senescent cells release a broad range of inflammatory cytokines, chemokines, matrix-remodeling enzymes, and growth factors, which can reinforce their own senescent state and induce secondary senescence in neighboring cells. Through this paracrine mechanism, senescence signals can propagate among different cardiac cell populations, thereby amplifying myocardial inflammation, extracellular matrix remodeling, and functional deterioration after DOX exposure [58,59].

The interaction between senescent cells and immune cells further contributes to the persistence of cardiac inflammation. SASP factors, including IL-1β, IL-6, TNF-α, and CCL2, regulate immune-cell recruitment and activation, while prolonged inflammatory signaling may impair effective resolution of tissue injury. In the context of DOX-induced cardiac damage, altered macrophage responses and sustained inflammatory activation may promote the accumulation of senescent cells and establish a self-reinforcing inflammatory microenvironment [28,47,60,61]. Collectively, these findings highlight that DOX-induced cardiac senescence is not restricted to individual cell types but involves coordinated interactions among regenerative cells, senescent cells, and immune components, ultimately contributing to progressive cardiac remodeling and dysfunction.

Importantly, the senescence-inducing effects of doxorubicin are not restricted to cardiac tissues. Although the present review focuses on cardiac senescence due to its clinical relevance and the particular vulnerability of the heart, evidence indicates that DOX can induce systemic senescence-like alterations in multiple organs. Increases in senescence-associated markers were observed across different tissues in a DOX-induced aging mouse model, supporting the concept that DOX accelerates organismal aging rather than causing an exclusively cardiac phenotype [62]. These findings indicate that DOX-induced cardiac senescence represents a component of a broader therapy-induced aging phenotype, while the heart may exhibit particularly pronounced vulnerability because of its high metabolic demand, mitochondrial dependence, and limited regenerative capacity.

## 3. Mechanisms of DOX-Induced Cardiac Senescence

**Figure 4:**
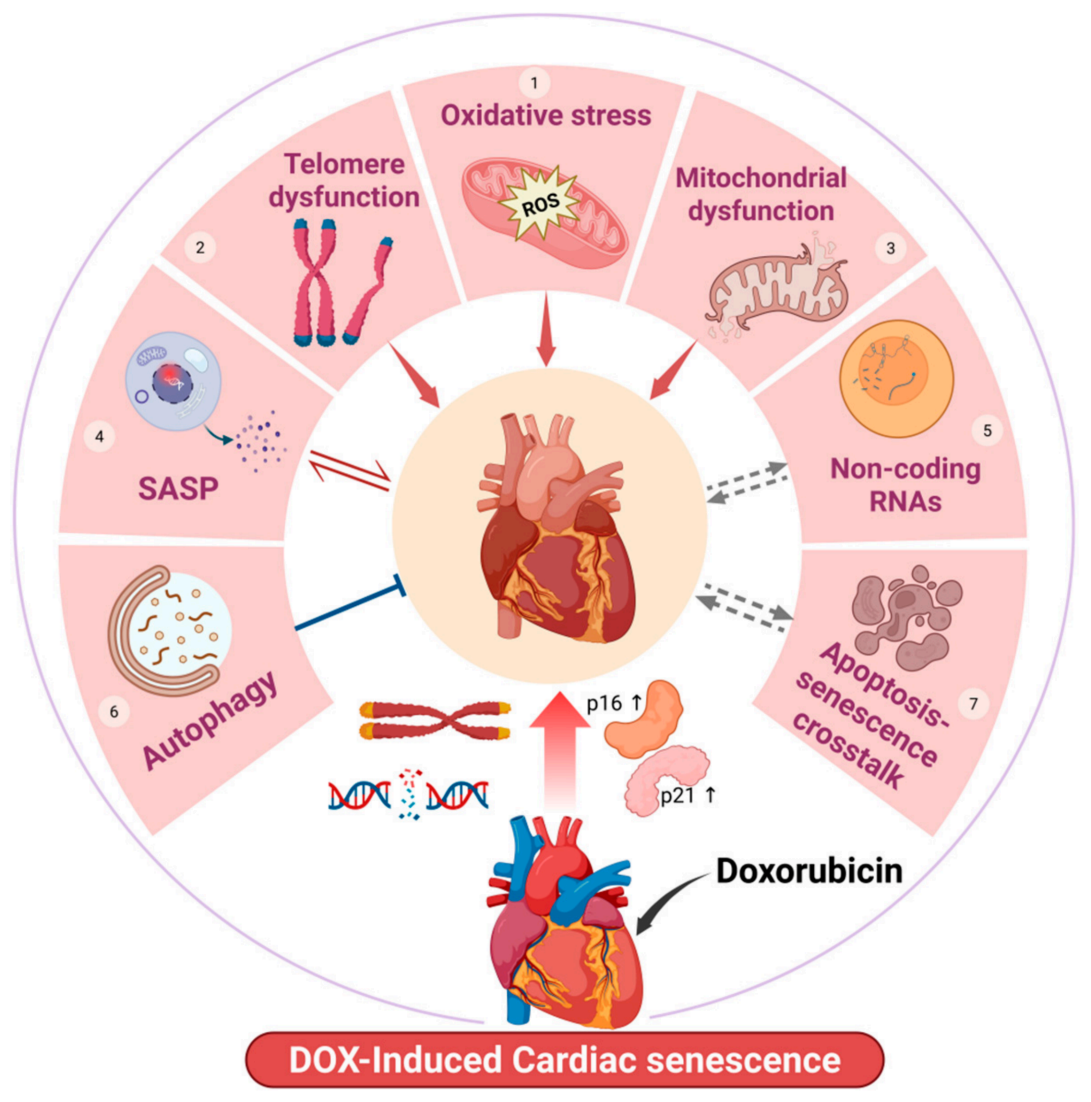
DOX-induced cardiac senescence involves multiple mechanisms, mainly including the following: (1) Oxidative stress: ROS-mediated redox imbalance represents a key mechanism. (2) Telomere dysfunction: DOX disrupts telomere homeostasis through telomere shortening, reduced telomerase activity, and TRF1/TRF2 downregulation, thereby activating the DNA damage response (DDR). (3) Mitochondrial dysfunction: DOX perturbs mitochondrial fission–fusion dynamics and metabolic function, thereby contributing to cardiac senescence. (4) SASP: low-dose DOX induces the senescence-associated secretory phenotype (SASP), whereas targeting SASP alleviates DOX-induced cardiac senescence. (5) Non-coding RNAs: circ-Foxo3, lincRNA-p21, miR-199a-3p, and other non-coding RNAs modulate cardiac senescence through downstream genes and signaling pathways. (6) Autophagy: activation of autophagy and mitophagy may mitigate DOX-induced cardiac senescence by restoring cellular and mitochondrial quality control. (7) Apoptosis–senescence crosstalk: high-dose DOX preferentially induces apoptosis, whereas lower-dose exposure promotes senescent growth arrest and may lead to delayed cell death. Solid red arrows indicate mechanisms that promote cardiac senescence; the red bidirectional arrow indicates reciprocal reinforcement between SASP signaling and senescence; the blue blunt-ended line indicates the protective effect of autophagy/mitophagy activation; and gray dashed bidirectional arrows indicate context-dependent regulatory relationships. Created in BioRender. Zhang, Z. (2026) https://BioRender.com/czrjr33.

Although DOX induces distinct senescence phenotypes in different cardiac cell populations, accumulating evidence indicates that these cellular responses are not independent events. Rather, the pathological alterations observed in cardiomyocytes, fibroblasts, endothelial cells, vascular smooth muscle cells, and other cardiac cells converge on several conserved molecular pathways that collectively drive cardiac senescence. These shared mechanisms include oxidative stress, telomere dysfunction, mitochondrial dysfunction, senescence-associated secretory phenotype (SASP) signaling, non-coding RNA dysregulation, impaired autophagy, and apoptosis (Figure 4). Therefore, after discussing the cell type-specific responses to DOX, this section shifts the focus to the canonical molecular mechanisms underlying DOX-induced cardiac senescence, thereby providing an integrated framework for understanding disease pathogenesis and identifying potential therapeutic targets.

### 3.1. Oxidative Stress

Oxidative stress, characterized by an imbalance between reactive oxygen species (ROS) generation and antioxidant defense systems, is an early and critical event in DOX-induced cardiac senescence. DOX undergoes one-electron reduction to form semiquinone radicals, which subsequently react with oxygen to generate ROS. Iron-dependent redox cycling and impaired antioxidant capacity further promote ROS accumulation, resulting in oxidative damage and activation of stress-responsive pathways [63,64].

Accumulating evidence indicates that oxidative stress functions as an upstream driver of DOX-induced senescence rather than merely a consequence of cardiac injury. Maejima et al. demonstrated that low-dose DOX exposure induced premature senescence in cardiomyocytes, characterized by increased senescence-associated β-galactosidase activity and persistent growth arrest without immediate apoptosis [19]. Excessive ROS accumulation subsequently promotes DNA damage responses and inflammatory activation, facilitating the establishment of stable senescent phenotypes [64].

Oxidative stress also interacts with inflammatory and regulatory networks to amplify cardiac senescence. DOX-induced activation of the C5a/C5aR axis increases ROS accumulation and inflammatory cytokine production, whereas C5aR inhibition attenuates oxidative stress and senescence-associated changes [36]. In addition, lincRNA-p21 has been implicated in DOX-induced cardiac senescence, with its inhibition reducing oxidative stress and senescence through regulation of the Wnt/β-catenin pathway [35]. Therefore, oxidative stress represents a central initiating mechanism that links DOX exposure to cardiac senescence.

### 3.2. Telomere Dysfunction

Telomeres maintain chromosome-end stability through their association with shelterin proteins and prevent inappropriate activation of DNA damage responses (DDRs). Telomere shortening or dysfunction is a well-established hallmark of cellular aging, as loss of telomere protection activates persistent DDR signaling, leading to irreversible cell-cycle arrest and senescence-associated phenotypes in cardiovascular tissues [65]. Beyond replicative aging, pathological stressors can induce premature telomere dysfunction and accelerate cellular senescence.

Accumulating evidence indicates that DOX induces cardiac senescence partly through disruption of telomere-associated structures. Spallarossa et al. demonstrated that DOX exposure induced either senescence or apoptosis in neonatal rat cardiomyocytes depending on stress intensity. Low-dose DOX decreased the expression of telomere-binding proteins TRF1 and TRF2, increased senescence-associated β-galactosidase activity, and promoted chromosomal instability, whereas higher doses caused more severe telomere damage and apoptotic cell death [5]. These findings suggest that impaired telomere protection, rather than simple telomere shortening, represents an early determinant of cardiomyocyte fate following DOX exposure.

Consistently, preservation of telomere-associated pathways can attenuate DOX-induced cardiac senescence. Testosterone was shown to counteract DOX-induced cardiomyocyte senescence by restoring TRF2 expression through the PI3K/Akt/eNOS signaling pathway, highlighting the importance of TRF2-mediated telomere protection in maintaining cardiomyocyte homeostasis [33]. In addition, DOX-induced telomere dysfunction has been observed in cardiac progenitor cells, resulting in impaired regenerative capacity and acquisition of senescent phenotypes [7]. Beyond chemotherapy-induced injury, aging-associated regulators such as SIRT6 also contribute to telomere maintenance and genomic stability, as SIRT6 deficiency promotes telomere shortening, DNA damage accumulation, and cardiac senescence [66]. These observations indicate that DOX-induced disruption of telomere integrity and associated DDR activation constitute important mechanisms linking chemotherapy exposure to cardiac cellular senescence.

### 3.3. Mitochondrial Dynamics and Dysfunction

Mitochondrial dynamics, mainly regulated by mitochondrial fusion and fission processes, are essential for maintaining mitochondrial quality control and metabolic homeostasis. Disruption of this balance can impair mitochondrial function and promote persistent cellular stress responses, which are increasingly recognized as important contributors to cellular senescence. Loss of the mitochondrial E3 ubiquitin ligase MARCH5 induces cellular senescence through dysregulation of DRP1- and MFN1-mediated mitochondrial remodeling, providing direct evidence linking mitochondrial dynamic imbalance to senescence induction [67].

Cardiomyocytes are highly dependent on mitochondrial oxidative metabolism to sustain continuous contractile activity; therefore, impaired mitochondrial remodeling has profound effects on cardiac homeostasis. Genetic disruption of mitochondrial dynamics in mouse hearts accelerates mitochondrial senescence and cardiac aging, demonstrating that maintaining mitochondrial dynamic balance is essential for preserving cardiac function [68].

Doxorubicin further disrupts mitochondrial homeostasis and contributes to cardiac senescence. Studies of DOX-induced cardiotoxicity demonstrate that DOX alters mitochondrial fusion–fission balance, impairs mitochondrial function, and triggers mitochondrial stress responses associated with senescence development [69]. Consistently, DOX and liposomal DOX induce senescence-associated phenotypes accompanied by mitochondrial alterations, including increased mitochondrial membrane potential, inflammatory activation, and upregulation of senescence markers such as p53 and SA-β-gal, suggesting that mitochondrial stress contributes to the establishment of DOX-induced cardiac senescence [4]. Furthermore, restoration of mitochondrial homeostasis provides protective effects against DOX-induced cardiac injury, as luteolin alleviates DOX-induced cardiac dysfunction by reducing DRP1-associated mitochondrial abnormalities and preserving mitochondrial integrity [34].

Beyond initiating mitochondrial injury, persistent mitochondrial dysfunction may also participate in the maintenance of the senescent phenotype. Kotla et al. demonstrated that chemotherapy-associated stress induced NAD^+^ depletion and mitochondrial dysfunction through ERK5 S496 phosphorylation-mediated PARP activation, establishing a nucleus–mitochondria positive feedback loop that promoted a persistent pro-inflammatory senescent phenotype [70]. Together, these observations indicate that mitochondrial dynamic imbalance and dysfunction represent important mechanisms linking DOX exposure to cardiac cellular senescence and provide a mechanistic basis for targeting mitochondrial homeostasis in DOX-induced cardiac senescence.

### 3.4. Senescence-Associated Secretory Phenotype (SASP) and Inflammatory Signaling

The senescence-associated secretory phenotype (SASP) is a key functional feature of senescent cells, characterized by the secretion of inflammatory mediators, growth factors, and matrix-remodeling molecules. SASP not only reflects the senescent state but also actively reinforces inflammatory signaling and promotes senescence propagation through paracrine effects. Nuclear factor-κB (NF-κB) is a central regulator of SASP-associated inflammatory responses [3].

In doxorubicin-induced cardiac senescence, inflammatory activation contributes to the establishment and maintenance of the senescent phenotype. Studies demonstrated that DOX and liposomal DOX induced cellular senescence accompanied by enhanced NF-κB activation, inflammatory responses, and increased senescence markers, indicating that inflammatory signaling is an integral component of DOX-induced senescence [4]. Furthermore, recent studies revealed that DOX-induced SASP-positive cardiomyocytes promote secondary senescence in neighboring cardiomyocytes through paracrine signaling, while depletion of these senescent cells alleviates DOX-related cardiotoxicity. Modulation of miR-199a-3p also reduces SASP production and suppresses both intracellular and paracrine senescence propagation, highlighting the contribution of SASP regulation to DOX-induced cardiac senescence [10,71].

These findings indicate that SASP-mediated inflammatory signaling serves as an important mechanism linking DOX-induced cellular stress to persistent cardiac senescence by amplifying inflammatory responses and facilitating senescence spreading within the myocardium.

### 3.5. Non-Coding RNA

Non-coding RNAs (ncRNAs), including microRNAs (miRNAs), long non-coding RNAs (lncRNAs), and circular RNAs (circRNAs), regulate gene expression through transcriptional and post-transcriptional mechanisms and have emerged as important modulators of doxorubicin-induced cardiac injury and senescence [72]. Unlike direct mediators of cellular damage, ncRNAs function as regulatory molecules that modulate senescence-associated signaling pathways, thereby influencing the initiation and progression of the senescent phenotype.

Several ncRNAs have been identified as positive regulators of DOX-induced cardiac senescence. Circ-Foxo3 is increased in aged cardiac tissues and DOX-induced aging models, and its overexpression aggravates DOX-associated cardiomyopathy, whereas circ-Foxo3 inhibition attenuates senescence-related cardiac injury [73]. Similarly, DOX exposure induces lincRNA-p21 expression, which promotes cardiomyocyte senescence through regulation of the Wnt/β-catenin signaling pathway; inhibition of lincRNA-p21 alleviates senescence-associated alterations in cardiomyocytes [35]. In addition, miR-34a contributes to DOX-induced senescence by targeting the phosphatase 1 nuclear targeting subunit (PNUTS), thereby promoting senescence progression in cardiomyocytes [74].

Conversely, several ncRNAs exhibit protective effects against DOX-induced cardiac senescence. Cardiac-specific overexpression of miR-199a-3p promotes cardiomyocyte cell-cycle reentry and proliferation, whereas its downregulation following DOX exposure contributes to impaired regenerative capacity and cardiac senescence [10]. Furthermore, exosomal lncRNA-NEAT1 derived from MIF-treated mesenchymal stem cells attenuates DOX-induced cardiac senescence by sponging miR-221-3p and activating Sirt2 signaling [75].

Overall, these findings indicate that ncRNAs represent an important regulatory layer in DOX-induced cardiac senescence by integrating senescence-associated signaling networks and modulating cardiomyocyte fate.

### 3.6. Autophagy

Autophagy is a critical cellular quality-control mechanism that maintains proteostasis and organelle homeostasis through the degradation and recycling of damaged cellular components. In the aging heart, impaired autophagic capacity contributes to defective quality control, accumulation of cellular damage, and progressive cardiac dysfunction. Studies demonstrated that age-associated decline in autophagy is closely linked to cardiac aging and functional deterioration [76]. Consistently, MIF deficiency aggravated age-related cardiac remodeling and dysfunction through dysregulation of autophagy, highlighting the essential role of autophagic homeostasis in maintaining cardiac function during aging [77].

Doxorubicin disrupts autophagic homeostasis and contributes to the development of senescence-associated cardiac injury. Recent evidence indicates that altered autophagy regulation represents an important component of DOX-induced cardiotoxicity, with both insufficient and dysregulated autophagic responses influencing disease progression [11]. Mechanistically, activation of autophagy has been shown to attenuate DOX-induced senescence. Cepharanthine reduced DOX-induced cellular senescence by activating autophagy through mTOR pathway regulation, while Sirt3-mediated autophagy activation alleviated DOX-induced senescence through inhibition of the PI3K/AKT/mTOR signaling pathway [78,79].

Beyond general autophagy, selective mitophagy plays a crucial role in maintaining mitochondrial quality control during cardiac aging. Parkin overexpression improved age-related cardiac remodeling and dysfunction by enhancing TBK1-associated mitophagy, emphasizing the importance of mitochondrial-selective autophagy in preserving cardiac homeostasis [80]. Thus, impaired autophagic regulation represents an important mechanism linking DOX-induced cellular stress, defective organelle quality control, and cardiac senescence.

### 3.7. Apoptosis

Apoptosis contributes to doxorubicin (DOX)-induced cardiac injury by promoting cardiomyocyte loss and impairing cardiac repair capacity. Increasing evidence indicates that DOX-induced cardiac damage does not exclusively result in apoptosis but may also drive cellular senescence, with cell fate determined by the intensity and duration of stress responses. In rat neonatal cardiomyocytes, DOX induced either apoptosis or senescence through regulation of telomere-binding factors TRF1 and TRF2, suggesting that telomere dysfunction contributes to the balance between cell death and senescent growth arrest [5].

DOX-induced apoptosis also affects cardiac regenerative potential. In human cardiac progenitor cells, DOX exposure caused senescence and functional impairment, reducing their proliferative and reparative capacity [7]. Consistently, anthracycline cardiomyopathy was associated with depletion of the cardiac progenitor cell pool, linking progenitor cell loss to impaired myocardial regeneration and progressive cardiac dysfunction [81]. Moreover, accelerated cardiomyocyte senescence contributes to late-onset DOX cardiotoxicity, indicating that persistent cellular damage and apoptotic loss promote persistent cardiac dysfunction [9].

## 4. Intervention Strategies Targeting DOX-Induced Cardiac Senescence

Accumulating evidence suggests that doxorubicin-induced cellular senescence represents an important pathological process contributing to cardiotoxicity [4,47,82]. In particular, increased senescence markers have been identified in the left ventricles of patients with DOX-induced cardiotoxicity, with more pronounced senescence observed in severe cases [82]. Collectively, these findings establish cellular senescence as a key pathological mechanism underlying DOX-induced cardiac injury and provide a strong rationale for developing senescence-targeted therapeutic strategies. The following section therefore summarizes current interventions that alleviate DOX-induced cardiac senescence and discusses their underlying mechanisms and therapeutic potential.

### 4.1. Holistic Non-Pharmacological Interventions

#### 4.1.1. Dietary and Nutritional Interventions

Emerging evidence indicates that nutritional status influences the myocardial response to doxorubicin (DOX). A high-fat diet (HFD) exacerbates DOX-induced cardiac injury by disrupting myocardial lipid metabolism, mitochondrial function, and fatty acid oxidation, whereas switching to a low-fat diet before and during DOX treatment partially reverses these detrimental changes and improves cardiac function [83,84]. Although these studies primarily evaluated cardiotoxicity rather than cellular senescence, they identified metabolic dysregulation as an important determinant of susceptibility to DOX-induced cardiac injury.

Recent evidence further suggests that lipid metabolic homeostasis and bioactive dietary compounds may influence the myocardial response to DOX. Among specific dietary lipids, docosahexaenoic acid (DHA), an omega-3 polyunsaturated fatty acid, attenuated DOX-induced cytotoxicity, reactive oxygen species production, and inflammatory NF-κB/iNOS/NO signaling in H9c2 cardiac cells [85]. Rosmarinic acid reduced DOX-induced cellular senescence by regulating the 14-3-3/FoxO1 signaling axis [86], whereas Fruitflow attenuated DOX-induced toxicity in H9c2 cells and improved high-fat diet-associated cardiac dyslipidemia in rats [87]. Together, these findings suggest that nutritional status, specific dietary lipids, and bioactive dietary compounds may modulate susceptibility to DOX-induced cardiac injury, although direct evidence regarding cellular senescence remains predominantly preclinical.

#### 4.1.2. Physical Exercise Training

Exercise training is increasingly recognized as an effective non-pharmacological intervention for reducing cardiovascular complications associated with doxorubicin (DOX) chemotherapy. A recent review summarized consistent evidence that exercise preserves cardiac function through multiple mechanisms, including improvement of mitochondrial function, attenuation of oxidative stress, maintenance of calcium homeostasis, suppression of apoptosis, and reduction in myocardial fibrosis [88]. Complementing these observations, two independent meta-analyses of rodent models further confirmed that aerobic exercise significantly preserves cardiac function and attenuates DOX-induced cardiac dysfunction across diverse experimental settings, providing robust preclinical evidence for exercise-mediated cardioprotection [89,90].

Mechanistic studies have begun to identify the molecular pathways underlying these protective effects. Exercise-induced upregulation of ADAR2 activates endogenous cardioprotective signaling through the miR-34a/SIRT1 axis [91], whereas exercise also improves mitochondrial metabolism and iron homeostasis during DOX treatment [92,93]. More recently, exercise was shown to attenuate DOX-induced cardiac immune dysregulation by regulating B-cell responses, highlighting immune modulation as an additional mechanism contributing to exercise-mediated cardioprotection [94].

Although current studies have primarily focused on DOX-induced cardiotoxicity rather than cellular senescence, exercise consistently modulates mitochondrial function, metabolic homeostasis, and immune responses, all of which are closely associated with the development of DOX-induced cardiac senescence. These findings support exercise as a promising adjunctive strategy for delaying premature cardiac senescence, although direct evidence linking exercise to the suppression of cardiomyocyte senescence remains limited.

### 4.2. Mechanism-Targeted Pharmacological Agents

Studies indicate that therapeutic interventions targeting upstream regulators of cellular senescence can attenuate DOX-induced cardiac senescence by modulating oxidative stress, mitochondrial homeostasis, inflammatory signaling, and cell cycle regulation. Rather than directly eliminating senescent cells, these approaches primarily interfere with the molecular events associated with senescence initiation and progression.

Restoration of SIRT1-dependent signaling has emerged as a common anti-senescent mechanism. In an aging-related cardiac model, berberine attenuated cardiac senescence through activation of the Klotho/SIRT1 axis [95]. Although this study was not performed in a DOX model, it provides supportive evidence that Klotho/SIRT1 signaling may represent a relevant senescence-regulatory pathway. Similarly, testosterone antagonized DOX-induced cardiomyocyte senescence through androgen receptor-dependent activation of the PI3K/AKT/NOS3 pathway, leading to preservation of telomere repeat-binding factor 2 (TRF2), a critical regulator of telomere integrity and cellular lifespan [33]. Consistent with these findings, roflumilast suppressed DOX-induced cardiomyocyte senescence through SIRT1 activation while reducing inflammatory responses [96].

Maintenance of mitochondrial homeostasis and cellular metabolism represents another important therapeutic strategy. CR6-interacting factor 1 (CRIF1) overexpression alleviated DOX-induced myocardial senescence by preserving mitochondrial function through inhibition of peroxidasin (PXDN) [97]. In addition, Semaglutide attenuated DOX-induced cardiac dysfunction by improving BNIP3-mediated mitochondrial dysfunction via PI3K/AKT signaling [98], whereas inhibition of CD38 preserved intracellular NAD homeostasis and mitochondrial energetics [99]. Likewise, 5-Oxoproline attenuated DOX-induced cardiac dysfunction by restoring glutathione metabolic homeostasis through the 5-oxoproline/OPLAH axis, suggesting that correction of metabolic disturbances may represent a promising upstream therapeutic strategy while simultaneously enhancing the antitumor efficacy of DOX [100], and Tirzepatide protected against DOX-induced cardiac injury by preventing HRD1-mediated Nrf2 ubiquitination and degradation, thereby reducing oxidative stress and cardiomyocyte apoptosis [101]. Although these studies primarily focused on cardiotoxicity rather than cellular senescence, they identify mitochondrial dysfunction, metabolic disturbance, and oxidative stress as potential upstream therapeutic targets relevant to senescence initiation.

Beyond pharmacological compounds, non-coding RNA and exosome-based therapeutic strategies have also shown anti-senescent potential. Cardiac-specific overexpression of miR-199a-3p promoted cardiomyocyte cell cycle re-entry and proliferation, thereby attenuating cardiac senescence through both intracellular and paracrine mechanisms [10]. Likewise, exosomal lncRNA-NEAT1 derived from MIF-pretreated mesenchymal stem cells protected against DOX-induced cardiac senescence by sponging miR-221-3p, leading to activation of SIRT2, highlighting the therapeutic potential of exosome-based interventions [75].

Drug repurposing may provide potential strategies for limiting long-term therapy-induced senescence. In juvenile DOX-exposed mice, aspirin alleviated systemic late adverse effects and reduced the accumulation of senescence markers in multiple tissues. In DOX-induced senescent fibroblasts, aspirin also reduced p53 and p21 accumulation and interfered with senescent-cell survival by decreasing Bcl-xL expression [102]. However, its cardiac-specific anti-senescence effects remain to be established.

### 4.3. Senescence-Targeted Therapeutic Strategies

#### 4.3.1. Senomorphic Strategies

Metformin mitigated DOX-induced senescence and hyper-inflammatory responses by reducing SASP factor secretion and adhesion molecule expression through inhibition of the JNK and NF-κB pathways, suggesting its potential role in preventing DOX-associated vascular aging and cardiac dysfunction [47]. Natural compounds have also emerged as potential senomorphic agents. A standardized extract of *Salvia haenkei* (Haenkenium, HK) reduced aging and DOX-induced senescence in mice, accompanied by decreased inflammatory and fibrotic responses. Mechanistically, luteolin, the major flavonoid component of HK, disrupted the p16^INK4a^-CDK6 interaction and suppressed senescence progression, highlighting a strategy to modulate senescent phenotypes without eliminating senescent cells [103].

Targeting inflammatory signaling represents an effective strategy for suppressing senescence progression. Redd1 was identified as a downstream effector of p38 MAPK that promoted cardiomyocyte senescence through NF-κB activation. Silencing Redd1 inhibited p38 MAPK-mediated p65 phosphorylation and nuclear translocation, thereby preventing DOX-induced cardiac senescence [104]. Consistently, Honokiol alleviated DOX-induced senescence in H9c2 cardiomyocytes by suppressing TXNIP-mediated NLRP3 inflammasome activation, accompanied by reduced SA-β-gal staining and decreased expression of p16^INK4a^ and p21 [37].

However, the therapeutic efficacy of senomorphic intervention requires further validation. In dynamically engineered heart tissues and human cardiomyocytes, DOX induced senescence-associated alterations similar to those observed in patients with cardiotoxicity. Although the senomorphic compounds AICAR and resveratrol reduced senescence-associated markers, they did not fully restore contractile function, suggesting that modulation of senescence pathways alone may not be sufficient to completely prevent DOX-induced cardiac dysfunction [82].

#### 4.3.2. Senolytic Strategies

Persistent cardiomyocyte senescence contributes to cardiac dysfunction and adverse remodeling through sustained SASP signaling, providing a strong rationale for senolytic therapy [105]. Accordingly, selective elimination of senescent cells has emerged as a promising strategy for mitigating DOX-induced cardiac senescence. In DOX-treated mice, pharmacological senolysis with Navitoclax significantly reduced senescence and cardiotoxicity markers, restored cardiac function, and delayed cardiac senescence, providing direct evidence that clearance of senescent cells can alleviate chemotherapy-induced cardiotoxicity [106].

Mechanistically, DOX-induced senescent cardiomyocytes acquire a pronounced SASP and propagate senescence to neighboring cardiomyocytes through exosome-mediated signaling. Selective depletion of these SASP-producing cardiomyocytes using the Bcl-2 inhibitor ABT-199 partially attenuated DOX-induced cardiac injury, highlighting SASP-positive senescent cardiomyocytes as critical therapeutic targets for limiting the propagation of cardiac senescence [71]. Consistently, the senolytic combination dasatinib plus quercetin rejuvenated DOX-treated human cardiac organoids by reducing oxidative stress, DNA damage, SASP, and senescence markers while restoring cardiomyocyte proliferation and contractile function, providing translational evidence supporting senolytic therapy in human cardiac models [107].

Beyond currently available senolytic agents, several novel therapeutic targets have recently been identified. Pharmacological inhibition of USP7 selectively induced apoptosis of senescent cells through restoration of p53 signaling and effectively reduced DOX-induced senescence and SASP in vivo [108]. Although these findings support USP7 as a potential senolytic target in DOX-induced systemic senescence, its efficacy in cardiac-specific models remains to be established. Furthermore, impaired Ca^2+^ transfer at mitochondria-endoplasmic reticulum contact sites (MERCSs) represents a specific vulnerability of therapy-induced senescent cells, and targeting this pathway produced potent senolytic effects in both in vitro and in vivo models [109]. In parallel, impaired autophagy has been identified as an upstream driver of cardiomyocyte senescence in both aging and DOX-treated hearts. Restoration of autophagy alleviated senescence, whereas senolytic treatment with ABT-263 reduced the accumulation of senescent cardiomyocytes and improved cardiac function, highlighting complementary strategies for targeting cardiac senescence [110].

### 4.4. MSC-Derived Paracrine and Extracellular Vesicle Approaches

Mesenchymal stem cell (MSC)-derived paracrine factors and extracellular vesicles have been explored as potential approaches for alleviating DOX-induced cardiac injury. Rather than direct cardiomyocyte replacement, their protective effects are primarily attributed to the transfer of bioactive molecules that regulate cellular stress responses, mitochondrial homeostasis, and senescence-associated pathways.

Experimental studies indicate that MSC-derived factors can attenuate DOX-induced senescence-associated phenotypes. MSC treatment reduced senescence markers and improved cardiomyocyte viability through modulation of VEGF/Notch/TGF-β signaling and miR-34a-associated pathways [111,112]. In addition, extracellular vesicles from MIF-preconditioned MSCs protected against DOX-induced cardiac senescence by delivering lncRNA-NEAT1, which suppressed miR-221-3p and enhanced Sirt2 signaling [75]. MSC-derived secretomes have also been shown to preserve mitochondrial metabolism and energy production in DOX-exposed cardiac cells [113]. Although these findings support the potential of MSC-derived approaches in modulating cardiac senescence, their therapeutic relevance remains largely preclinical and requires further validation.

### 4.5. Traditional Chinese Medicine

Traditional Chinese Medicine (TCM)-derived formulations and bioactive compounds have been increasingly investigated as potential interventions against DOX-induced cardiotoxicity. Although most studies have evaluated cardiac injury rather than classical senescence phenotypes, emerging evidence suggests that TCM may attenuate DOX-associated cardiac senescence by targeting several senescence-related processes, including mitochondrial dysfunction, oxidative stress, autophagy imbalance, regulated cell death, and inflammatory signaling.

Mitochondrial protection represents a major mechanism underlying the cardioprotective effects of TCM. Shenmai injection preserved mitochondrial homeostasis and reduced DOX-induced oxidative injury through regulation of AMPK and PI3K/Akt/GSK-3β signaling pathways [114]. Similarly, Qishen granules and Linggui Zhugan decoction improved mitochondrial function and reduced oxidative stress through activation of the SIRT3/SOD2 and AMPK–FOXO3a pathways, respectively [115,116]. Fermented Cordyceps sinensis also protected against DOX cardiotoxicity by improving antioxidant capacity and cardiac energy metabolism through AMPK-related mechanisms [117]. These findings indicate that TCM may alleviate upstream pathological events that contribute to cardiac senescence.

Importantly, some TCM-derived compounds have demonstrated more direct effects on senescence-associated alterations. Gastrodin reduced DOX-induced cardiomyocyte senescence by decreasing SA-β-gal activity and p16/p21/p53 expression, partly through regulation of AMPK/mTOR/4EBP1-dependent autophagy [118]. Polysaccharides derived from Panax ginseng and Codonopsis pilosula attenuated DOX-induced myocardial senescence by suppressing senescence-associated inflammatory responses through modulation of the TLRs/SAPS2/PPP6C/NF-κB pathway [119].

Furthermore, TCM-derived active components may also influence other stress-responsive pathways that contribute to the progression of DOX-induced cardiac senescence. Ginsenoside Rb1 attenuated DOX-induced cardiac injury by reducing oxidative stress and suppressing excessive autophagy and ferroptosis [120], while Sheng-Mai-Yin and Danggui Buxue decoction inhibited DOX-induced ferroptosis and ZBP1-mediated PANoptosis, respectively [121,122].

From a translational and pharmacological perspective, the TCM interventions discussed above differ considerably in their degree of chemical characterization. Gastrodin and ginsenoside Rb1 are chemically defined compounds, allowing their molecular targets and dose-dependent effects to be investigated more precisely [118,120]. In contrast, formulations such as Shenmai injection, Qishen granule, and Linggui Zhugan decoction contain multiple constituents. Modern mass spectrometry and chromatographic fingerprinting studies have characterized multiple ginsenosides, ophiopogon-derived constituents, flavonoids, saponins, and other compounds in these formulations [123,124,125]. Pharmacokinetic studies further indicate that only a subset of these constituents achieves measurable systemic or tissue exposure and that different components exhibit distinct absorption and elimination profiles [126,127]. Therefore, their therapeutic effects may arise from a principal active constituent, the additive actions of multiple components, or interactions among component combinations. However, potential synergistic effects require direct experimental validation rather than inference from chemical profiling alone. Further studies should combine activity-guided fractionation, chemical standardization, pharmacokinetic analysis, and direct comparisons between isolated compounds and reconstructed mixtures. Identification of the principal active constituents may facilitate standardized dosing, target validation, and the development of more precisely defined pharmacological interventions.

Despite these advances, the active constituents and pharmacological basis of many TCM formulations remain incompletely defined, and direct evidence demonstrating delayed cardiac senescence remains limited. Future studies integrating active-component identification, chemical standardization, multi-omics approaches, pharmacokinetic evaluation, and validated senescence models are required to determine whether TCM directly targets cardiac senescence or primarily mitigates the upstream stress pathways that drive senescence.

## 5. Conclusions and Future Perspectives

Accumulating evidence indicates that DOX-induced cardiac injury is closely associated with premature cardiac senescence, which differs from physiological cardiac aging in its initiating stimuli and pathological progression. DOX-induced senescence involves multiple cardiac cell populations, including cardiomyocytes, fibroblasts, endothelial cells, vascular smooth muscle cells, progenitor cells, and immune-associated networks, collectively contributing to impaired cardiac homeostasis and pathological remodeling.

Recognition of senescence as a critical mediator of chemotherapy-associated cardiac aging has shifted therapeutic strategies from conventional cardioprotection toward senescence-oriented interventions. Emerging approaches, including senomorphic and senolytic therapies, mitochondrial and metabolic modulation, and MSC-derived extracellular vesicles, provide promising opportunities to delay or alleviate DOX-induced cardiac senescence. Traditional Chinese Medicine-derived formulations and bioactive compounds may also alleviate senescence-related cardiac injury by modulating mitochondrial dysfunction, oxidative stress, autophagy, and inflammatory signaling. However, direct evidence for their anti-senescence effects remains largely preclinical, and further studies are required to determine their therapeutic efficacy, safety, and clinical applicability.

Future research should focus on identifying clinically applicable biomarkers for early detection of DOX-induced cardiac senescence and clarifying cell-specific mechanisms that determine the fate of stressed cardiac cells. Further validation of senescence-targeted therapies, extracellular vesicle-based approaches, and bioactive compounds in clinically relevant models is required. Importantly, future interventions should selectively protect cardiac tissues while preserving the anticancer efficacy of DOX, thereby improving the therapeutic window of anthracycline chemotherapy.

## Data Availability

No new data were created or analyzed in this study. Data sharing is not applicable to this article. All data cited in this review are derived from publicly published literature.
